# Molecular characterization and evaluation of complex rearrangements in a case of ring chromosome 15

**DOI:** 10.1186/s13039-017-0339-z

**Published:** 2017-10-25

**Authors:** Stuti Tewari, Naznin Lubna, Raju Shah, Ahmed B. H. Al-Rikabi, Krati Shah, Jayesh Sheth, Frenny Sheth

**Affiliations:** 1 FRIGE’s Institute of Human Genetics, FRIGE House, Jodhpur Gam Road, Satellite, Ahmedabad, 380009 India; 2 Ankur Institute of Child Health, Ashram Road, Ahmedabad, 380009 India; 3 Institute of Human Genetics, Am Klinikum 1, 07747 Jena, Germany

**Keywords:** Ring chromosome 15, Array-comparative genomic hybridization (aCGH), Duplication/deletion, IGF-1R gene, Microcephaly

## Abstract

**Background:**

Ring chromosome 15 is a rare genetic entity. Only a few cases have been reported with characterization using molecular techniques. The clinical presentation is quite variable, as a result of differences in the breakpoints, haploinsufficiency of genes involved in deleted segment/s, level of mosaicism and ring instability resulting in a variability of rearrangement of genetic material.

**Case presentation:**

The proband, a 2 months old boy, presented with small head size and facial dysmorphism. On examination microcephaly, triangular face, small anterior frontanelle, micrognathia, hypotonia, unilateral simian crease, hypertelorism, umbilical hernia, micropenis with mild phimosis were noted. Karyotype revealed 46,XY,r(15)(p11.2q26). Array-comparative genomic hybridization (aCGH) and targeted gene sequencing for microcephaly was carried out for genotype phenotype correlation. Array-CGH detected a 2.8 Mb terminal deletion at 15q26.3 along with a 496 kb interstitial micro-duplication, encompassing the *IGF1R* gene, in the affected genomic region, which was otherwise missed on conventional karyotype.

**Conclusion:**

The present study highlights the importance of aCGH in not only delineating specific phenotypes through accurate genotypic correlation but also in detection and evaluation of ring chromosome with unexpected complex rearrangements.

**Electronic supplementary material:**

The online version of this article (10.1186/s13039-017-0339-z) contains supplementary material, which is available to authorized users.

## Background

Ring chromosome 15 is associated with a rare disorder that was first described by Jacobsen in 1966 [1]. Around 50 cases with r(15) have been reported till date, with only a few cases being comprehensively characterized using molecular cytogenetic techniques [2, 3]. Major clinical features include severe pre- and postnatal growth delay, microcephaly, triangular face, hypertelorism, intellectual disability, clinodactyly and brachydactyly of the fifth finger, small hands and feet, and cafe-au-lait spots [2, 4, 5]. Moreover, more severe phenotypes may include congenital heart defects, renal anomalies, hypotonia, hydrocephaly, retinal abnormalities, ear anomalies, behavioral disorders and speech delay [2]. The varying degree of phenotypic severity can be attributed to differences in the breakpoints, haploinsufficiency of genes involved in the deleted segments, level of mosaicism and ring instability resulting in a variability of other genetic material rearrangements [6, 7].

In the present case report, we attempted to do a precise genotype and phenotype correlation in a male proband with ring chromosome 15 using array comparative genomic hybridization (aCGH) and targeted gene sequencing. The report also aims to highlight the utility of aCGH in the detection and evaluation of complex rearrangements in atypical ring chromosomes. Furthermore, the case was studied by banding and molecular cytogenetics.

## Case presentation

The proband, a 2 months old boy, was born full term to 33-year-old non-consanguineous parents, by Caesarian section. The child had an APGAR score of ten with a birth weight of 2.8 kg. At the time of presentation, the proband was 2 months old; his length and weight were at the 3rd centile while the head circumference being significantly less than the 3rd centile, according to WHO (i.e.: 54 cm, 4.15 kg and 34 cm respectively). The proband had microcephaly, triangular face, small anterior frontanelle, micrognathia, hypertelorism unilateral simian crease, umbilical hernia and micropenis with mild phimosis. On neurological examination, the child presented hypotonia with oromotor delay. Ophthalmologic, auricular and cardiac examinations were normal. MRI brain revealed oligogyria with normal myelination in accordance with age suggesting the possibility of microcephaly with simplified gyral-pattern (MSG Group-1).

The proband was the second child of his parents (mother G2P2). His elder brother was apparently normal. The family history was unremarkable; the parents had a normal phenotype. The baby was referred to our centre for karyotyping and genetic counseling. Sample collection and written informed consent was obtained according to the regulations of the institutional ethics committee working according to the Helsinki declaration.

**Fig. 1:**
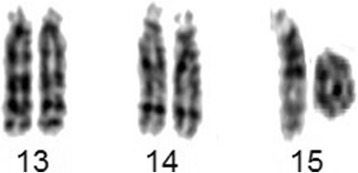
Partial karyotype showing ring chromosome 15, i.e. r(15)(p11.2q26)

**Fig. 2:**
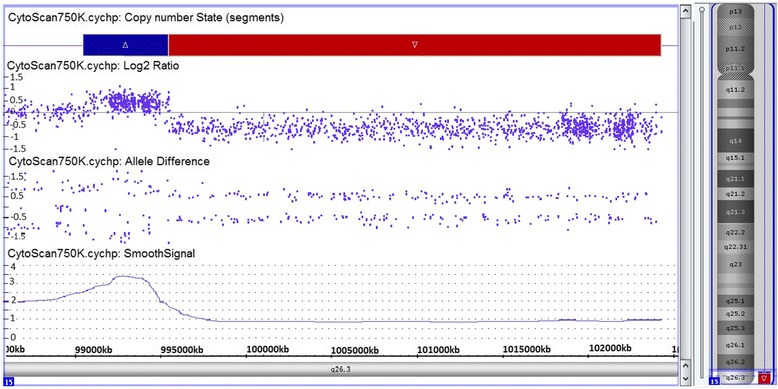
Array-CGH profile of the patient showing a 2.8 Mb loss at 15q26.3 and 496 kb gain at 15q26.3 i.e. arr[GRCh37] 15q26.3(99550797_102429040)×1,15q26.3(99049746_99546177)×3

**Fig. 3:**
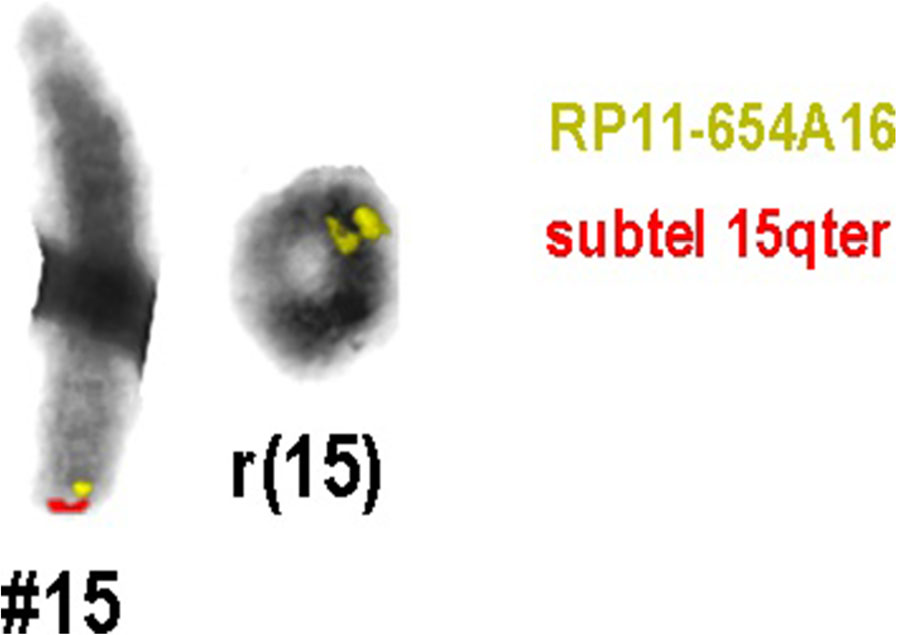
Partial karyotype showing comprehensive characterization of the chromosome ring15 in the proband’s karyotype and was identified as 46,XY,r(15)(p11.2q26),ish r(15)(p11.2q26)(RP11-654A16++)(subtel15qter-)

Chromosome analysis performed on proband’s 72-h lymphocyte cultures revealed an apparently stable non-mosaic ring chromosome 15, instead of one normal chromosome 15 [46,XY,r(15)(p11.2q26)] (Fig. 1). None of the 100 metaphases showed secondary aberrations. The karyotypes of the parents were normal. Array-CGH was carried out to characterize possible additional submicroscopic rearrangements associated with ring chromosome 15. DNA was extracted from the patient’s peripheral white blood cells using salting out method [8]. aCGH was performed by Affymetrix CytoScan™ 750 K array and obtained data was analyzed using Chromosome Analysis Suite (ChAS) based on the Human reference genome (GRCh37/hg19). It revealed according to ISCN 2016 [9] as arr[GRCh37] 15q26.3(99550797_102429040)×1,15q26.3(99049746_99546177)×3, indicating for a 496 kb gain encompassing *IGF1R* gene, and for a loss of 2.8 Mb, both in 15q26.3, and the latter encompassing genes like *MEF2A, CHSY1, ADAMTS17, ADLH1A3* (Fig. 2). The genomic imbalance was later confirmed by fluorescence in situ hybridization (FISH) as well. For FISH various probes were applied, like a microdissection derived probe for all acrocentric p-arms (midi54 [10]), the locus-specific probe RP11-654A16 in 15q26.3 (GRCh37: 99338019_99529036), and two commercially available probes (Abbott/Vysis, Wiesbaden, Germany) for subtelomeric region in 15qter and a centromere-specific probe for 15p11.1-q11.1 (Fig. 3). No other relevant genomic imbalance was found, however; by aCGH not addressable short arm of ring chromosome 15 was also lost, as visualized by probe midi54.

Both parents were also investigated using aCGH and by quantitative polymerase chain reaction (qPCR) to rule out the inheritance of either duplication or deletion in the proband using following primers:

-to address *LRR28* gene, exon 5 [for deleted region]
- 5′ TTG AAG CCA TTG GGT CTC TT 3′
- 5′ CCT GGA GGT AGG AAT TGC AG 3′
-to address *IGF1R* gene, exon 13 [for duplicated region]
- 5′ ATC AGC GAG AAT GTG TGT CC-3′
- 5′ TTG GCC TGG ACA TAG AAG AA 3′.

qPCR established a de novo origin of deletion and duplication in the proband.

Selective capture and sequencing of the protein coding regions of the genes of the microcephaly panel (Additional file 1) was also performed, wherein no pathogenic and likely pathogenic variants causative of the reported phenotype was detected.

## Discussion and conclusion

Ring chromosome 15 is a rare finding, generally occurring as a de novo event [11]. Here, we report one of the few cases of a ring chromosome 15 that have been characterized using molecular cytogenetic technique – FISH and aCGH, which not only helped determine the exact breakpoints, enabling precise genotype-phenotype correlation, but also offered an insight into the plausible mechanisms of ring formation. Simultaneously, targeted gene sequencing of the microcephaly panel ruled out the presence of other causative mutations that could have possibly given rise to microcephaly with simplified gyral-pattern (MSG Group-1).

The results of aCGH showed a 2.8 Mb terminal deletion at 15q26.3 along with a 496 kb interstitial micro-duplication in the adjacent region. The findings were further confirmed and refined for breakpoint in short arm by FISH analysis. Recent studies have discovered that a small proportion of ring chromosomes with terminally deleted segments are found to have a contiguous duplicated genomic segment [12, 13]. Knijnenburg et al. [12] and Rossi et al. [13] in their respective studies highlighted the presence and the underlying mechanisms of duplicated segments in ring chromosomes with terminal deletions and hypothesized that inverted duplication deletion rearrangements may have been stabilized by circularization that result into ring formation. Guilherme et al. [6] further concluded that phenotypic correlation in patients with ring chromosome cannot be done by assuming a simple deletion without excluding the detection of additional duplicated segments, thereby resulting in not just partial monosomy but also a partial trisomy [6]. They emphasized the importance of molecular genetic testing in detecting complex rearrangements like these, which are otherwise easily missed on conventional karyotyping. The present case, to the best of our knowledge, is only the second case of ring chromosome 15 with terminal deletion and a contiguous duplication at 15q26.3. The first one was reported by Rossi et al. in 2008 [13]. However, we could not determine the inverted/tandem nature of duplication due to its small size.

In general, clinical phenotypes of patients with ring chromosomes may be related with different factors, including gene haploinsufficiency, gene duplications, ring instability and level of mosaicism [6, 7]; also, loss of ring chromosomes may lead for some chromosomes to mosaic karyotypes with 45 chromosomes [14]. The proband under report presented with primary observation of small head size and facial dysmorphism. On examination microcephaly, triangular face, micrognathia, small anterior frontanelle, slanted skull, hypotonia, unilateral simian crease, hypertelorism, umbilical hernia, micropenis with mild phimosis were noteworthy. Many patients with ring chromosome 15 syndrome frequently present with pre- and postnatal growth retardation, developmental delay, microcephaly, craniofacial dysmorphism, hypertelorism, café-au-lait spots and brachydactyly [4, 5]. The specific pattern of microcephaly with simplified gyral-pattern observed in the present case is being reported for the first time.

Only a couple of genes have been mapped to date in the distal part of chromosome 15, 15q26.3, one of which is Insulin like Growth Factor 1 Receptor gene (*IGF-1R)*. Other morbid genes include *MEF2A* (*600660)*, ADAMTS17* (*607511)*, CERS3* (*615276)*, CHSY1* (*608183)*, ALDH1A3* (*600463) (DECIPHER database, https://decipher.sanger.ac.uk/). Apart from ring instability, marked growth deficiency in cases of ring chromosome 15 has been attributed to loss of *IGF-1R* [2, 3, 15–17]. Interestingly, a micro-duplication at 15q26.3 was detected in the current case, resulting in three copies of *IGF-1R,* which in turn has been linked to overgrowth and mental retardation [18]. However, the case under report had mild growth deficiency. Considering that the ring chromosome 15 was apparently stably transmitted during cell division, the plausible explanation for growth retardation could be the reduced level of expression of the *IGF1R* gene. The mechanisms responsible for the reduction of *IGF1R* expression are not clear but could have resulted from haploinsufficiency of a gene or genes within the deleted region that modifies *IGF1R* expression or the mechanism of gene silencing taking place due to a telomere position effect in which the 15p telomere silences nearby gene(s) on the q-arm or a spread of the heterochromatin-inactivated state of the centromere and short-arm (15p) DNA to the adjacent long arm (15q), also known as position effect variegation (PEV) [6, 19, 20]. In a recent paper Leffler et al. [19] proposed the possibility of *LRRK1* as being another growth regulating gene located at the distal region of 15q [19]. Few other authors too have questioned the role of *IGF-1R* gene as the sole growth determining factor [4, 15].

There is still a paucity of literature on ring chromosome 15 and further studies on genotype phenotype correlation are indicated. The case reported here, together with clinical and molecular findings highlights the importance of aCGH in detection and systematic evaluation of atypical ring chromosomes, especially in distinguishing rings with a duplication/deletion from those with a deletion only and thereby helping to delineate precise genotype-phenotype correlation. We would like to add that further expression studies of candidate genes located at 15q26.3 are essential to prove their biological significance in human growth and development.

## Additional file

Additional file 1: Microcephaly panel (a total of 69 genes) was analyzed. The list of genes is mentioned. (DOCX 11 kb)

## Abbreviations

aCGH: Array based comparative genomic hybridization
DNA: Deoxyribonucleic acid
FISH: Fluorescence In Situ Hybridization
MRI: Magnetic Resonance Imaging;
