# Supplementary material for: Molecular characterization and evaluation of complex rearrangements in a case of ring chromosome 15
Source: Mol Cytogenet. 2017 Oct 25;10:38. doi: 10.1186/s13039-017-0339-z (PMC5657133; doi:10.1186/s13039-017-0339-z)
Supplement: Additional file 1: — Microcephaly panel (a total of 69 genes) was analyzed. The list of genes is mentioned. (DOCX 11 kb) [file 13039_2017_339_MOESM1_ESM.docx]

**Supplementary information**

Microcephaly panel (a total of 69 genes) was analyzed. The list of genes is mentioned below.

*ARFGEF2, ORC4, ASPM, ORC6, ASXL3, PCNT, ATR, PHC1, ATRX, PLK4, CASC5, PNKP, CASK, PPP1R158, CDC6, QARS, CDK5RAP2, RAB18, CDK6, RAB3GAP1, CDKL5, RAB3GAP2, CDT1, RAD50, CENPE, RBBP8, CENPJ, RTTN, CEP135, SASS6, CEP152, SLC25A19, CEP63, SLC2A1, CRIPT, SLC9A6, DIAPH1 , STAMBP, DYRK1A, STIL, FOXG1, TBC1D20, IERIP1, TCF4, KATNB1, TRAPPC9, KIF11, TRMT10A, LIG4, TSEN2, MCPH1, TSEN34, MECP2, TSEN54, MED17, TUBGCP4, MFSD2A, TUBGCP6, MSMO1, UBE3A, NBN, WDR62, NDE1, XRCC4, NHEJ1, ZEB2, NIN, ZNF335, ORC1*
